# Physical Activity, Alcohol, and Cigarette Use in Urological Cancer Patients over Time since Diagnosis

**DOI:** 10.3390/healthcare12010059

**Published:** 2023-12-26

**Authors:** Bartosz Bogusz Adamczak, Zofia Kuźnik, Szymon Makles, Andrzej Wasilewski, Aureliusz Andrzej Kosendiak

**Affiliations:** 1 Student Scientific Association, Department of Physical Education and Sport, Wroclaw Medical University, 51-601 Wroclaw, Poland; 2 Department of Physical Education and Sport, Wroclaw Medical University, 51-601 Wroclaw, Poland

**Keywords:** urologic neoplasms, exercise, alcoholism, tobacco use disorder

## Abstract

Urological cancers represent a substantial global public health concern, exerting far-reaching effects on both individuals and their families. There is an urgent need to comprehensively understand the transformations in patients’ lifestyles and behaviors, given their critical role in the treatment process and overall well-being. This study, involving 128 urological cancer patients, aims to investigate changes in physical activity levels, problematic drinking behaviors assessed through the Alcohol Use Disorders Identification Test (AUDIT), and smoking habits assessed using the Fagerström Test for Nicotine Dependence (FTND) over four distinct time intervals over the subsequent three years from the time of diagnosis and among individuals diagnosed more than three years ago. The results reveal a significant decrease in physical activity levels between study intervals (*p* < 0.0001), declining from 69% to 45% between the first and second post-diagnosis assessments. Furthermore, the highest levels of problematic substance use, indicated by mean scores, were noted in the first year following diagnosis (AUDIT: 4.20, *p* = 0.01; FTND: 4.83, *p* = 0.08). Given the significant impact of physical activity on the prospects of recovery, it is imperative to delve more deeply into the factors contributing to this decline and devise targeted interventions for its improvement. In the context of substance use, it is essential to ascertain whether the initially high levels are a result of coping with the cancer diagnosis or represent a turning point at which patients modify their behaviors and cease their addiction. A more thorough understanding of this phenomenon would enhance the effectiveness of precisely focused interventions.

## 1. Introduction

Urinary bladder cancer ranks as the sixth most frequently diagnosed cancer in Poland, while kidney cancer is among the top 10 most commonly diagnosed cancers in the country [1]. Hence, it is apparent that urological cancers pose a significant healthcare concern in Poland. As supported by scientific literature, the quality of life for patients is not solely contingent upon their physical health but is also intricately linked to various facets of their existence [2]. The physical and psychological well-being of cancer patients is shaped by their lifestyle, which, to some extent, may play a role in the development or recurrence of cancer [3].

One determinant influencing the quality of life among individuals grappling with cancer is physical activity (PA), and an escalation in PA demonstrates a positive correlation with life satisfaction [4]. Current literature indicates that PA has many benefits for cancer patients. It alleviates the severity of treatment-related side effects, reduces fatigue, improves quality of life, positively impacts mental health, and improves aerobic capacity. In addition, it reduces the risk of cancer recurrence and mortality [5,6].

Additionally, the investigation encompassed an examination of the frequency of alcohol intake. Certain researchers have pointed to a declining trend in the utilization of this psychoactive substance [7]. Nonetheless, there exists compelling evidence that both alcohol usage and risky drinking behaviors are prevalent among individuals who have successfully recovered from cancer and those undergoing treatment [8]. It is crucial to emphasize that alcohol consumption is linked to heightened postoperative risks [9,10] and exacerbates the efficacy of cancer therapy [8]. These factors potentially carry implications for the outcomes of surgical interventions in patients afflicted with urological cancers. To achieve a more comprehensive understanding of the underlying mechanisms involved, it becomes imperative to identify the temporal patterns associated with alcohol consumption, a dimension that this study systematically explores.

A cancer diagnosis represents a crucial turning point in an individual’s life, with the potential to greatly impact their perception of life satisfaction. Elevated stress levels may lead to an increase in cigarette consumption as a coping mechanism [11]. Conversely, it could also serve as a valuable opportunity for making significant decisions, such as the decision to quit smoking. Research indicates that a substantial proportion of patients opt to quit smoking following a cancer diagnosis, though in cases of urologic cancer, this proportion is notably lower compared to other types of cancer [12]. Furthermore, the literature points to an elevated risk of urological cancer incidence and poorer prognoses among individuals who smoke [13].

Considering the noteworthy influence of non-health factors on life satisfaction, this research endeavor was undertaken to examine the engagement in PA and the consumption of alcohol and cigarettes, among individuals diagnosed with urologic cancer. The inclusion of PA, smoking, and alcohol intake as investigated variables for cancer patients was based on their acknowledged significance as fundamental lifestyle factors relevant to cancer survivorship [14,15,16]. A particular focal point of this study was the temporal aspect, specifically, the duration that had elapsed since the cancer diagnosis. This temporal dimension was emphasized to facilitate the inference of conclusions regarding the variability of the factors under investigation in relation to the duration of the disease.

## 2. Materials and Methods

### 2.1. Study Design and Participants

**Figure 1:**
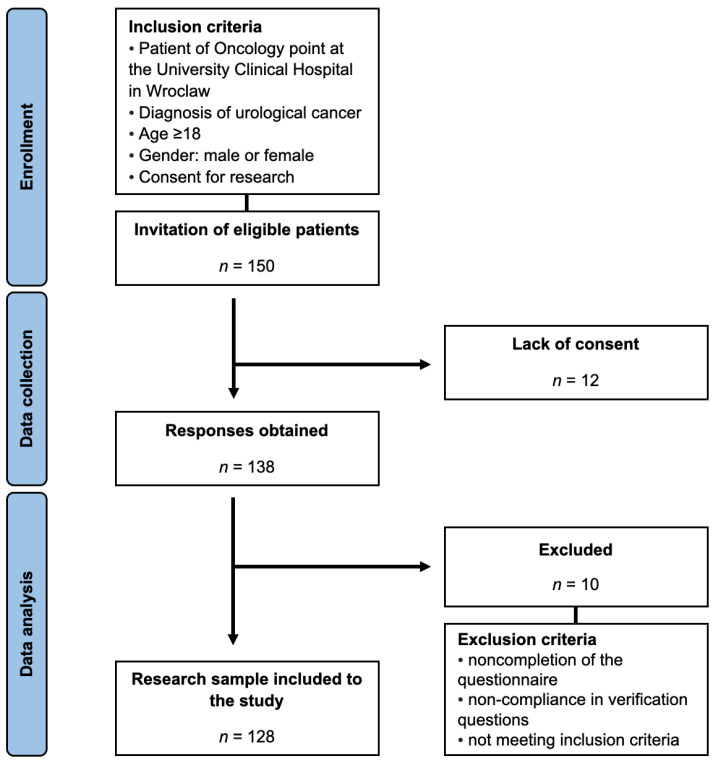
Study selection process.

Patients visiting the Oncology Center at the University Clinical Hospital in Wroclaw, diagnosed with urological cancers, were recruited for this study, which was conducted between February and July 2022. After informed consent was obtained, participants were presented with the research questionnaire. Data collection was carried out through the Computer Assisted Web Interview technique, ensuring complete anonymity. A detailed outline of the participant selection process, as well as the inclusion and exclusion criteria, is provided in Figure 1. 

The questionnaire encompassed the instruments described in subsequent sections and a segment aimed at gathering fundamental patient characteristics, including the time since their cancer diagnosis. This duration was categorized into four groups: within one year of diagnosis, between one and two years from diagnosis, between two and three years from diagnosis, and more than three years from diagnosis.

The study was designed as a cross-sectional study. Given that the facility where the study was conducted serves as one of the primary specialized clinics in the region for these types of conditions, it was assumed that the entire patient population would most accurately represent the characteristics of local individuals with urological cancers. To maximize representativeness, all patients meeting the inclusion criteria were invited to participate in the study.

With an annual prevalence of urological cancers in Poland estimated at around 10,000 cases [1], assuming a confidence level of 95% and a margin of error of 8%, the sample size was determined to be approximately 150 individuals—this number of patients was preliminarily invited to participate in the study.

### 2.2. International Physical Activity Questionnaire (IPAQ)

The IPAQ is a self-report questionnaire designed to assess PA levels. It consists of seven questions, with six inquiring about the frequency and duration of PA in the past week, categorized into walking, moderate, and vigorous intensity. The seventh question addresses the daily sitting time over the past week. To mitigate potential overestimation by respondents, all participants in the survey received training from our research team [17].

The weekly duration of each specific PA is multiplied by its corresponding metabolic equivalent of task (MET) value, where walking corresponds to 3.3 MET, moderate intensity to 4.0 MET, and vigorous intensity to 8.0 MET. The resultant score is expressed in MET-minutes per week (MET-m/w) [18].

The IPAQ is a questionnaire with established validity and reliability, demonstrated through research, including studies conducted within the Polish population [19,20].

### 2.3. Alcohol Use Disorders Identification Test (AUDIT)

The AUDIT questionnaire is a tool developed by the World Health Organization to assess harmful alcohol consumption. It comprises questions that universally and culture-independently address alcohol-dependent problematic behaviors and experiences encountered by individuals with alcohol-related issues. The questionnaire consists of 10 questions, with scores ranging from 0 to 4 points each. The cumulative score is interpreted according to the following criteria: a score of 0–7 suggests low-risk consumption, a score of 8–14 suggests medium-risk consumption, and a score of 15 or higher suggests possible alcohol dependence [21].

The AUDIT is a questionnaire with established validity and reliability, demonstrated through research, including studies conducted within the Polish population [22].

### 2.4. Fagerstrom Test for Nicotine Dependence (FTND)

The FTND is a tool commonly employed to assess the level of nicotine addiction. It consists of six questions pertaining to smoking-related behaviors. The questions with binary (yes/no) responses were scored on a scale ranging from 0 to 1, while multiple-choice questions were scored from 0 to 3. The resulting score determines the degree of nicotine dependence, with a prescribed categorization: 0–3 points indicating low dependence, 4–6 points indicating moderate dependence, and 7–10 points indicating high dependence [23].

The FTND has been recognized as a valid and reliable method for measuring nicotine dependence in the Polish population [24,25].

### 2.5. Statistical Analysis

In this study, Microsoft Excel version 16.77 (Redmond, WA, USA) was utilized for cleansing, curation, and computation of raw data. Statistical analysis, including the identification of significant relationships, was performed using Statistica 13 (Statsoft, Kraków, Poland). 

The data collected in the survey exhibited a non-normally distributed pattern as determined by the Shapiro–Wilk test. A descriptive analysis was employed encompassing frequency and percentage measures for qualitative variables, median and interquartile range (IQR) for PA, and mean values with 95% confidence intervals (95% CI)—to improve data comprehensibility—for the outcomes derived from AUDIT and FTND. To compare qualitative variables, the Chi-square test was utilized, and Crammer’s V effect size was computed. For the comparison of quantitative variables, the Kruskal–Wallis test was employed, along with the calculation of eta squared as the effect size measure. Moreover, a multivariable logistic regression model was built with Inactive IPAQ level as a dependent variable and the following confounders as independent variables: age, gender, place of residence, time since diagnosis, alcohol drinking, and cigarette smoking. All analyses were performed at a significance level of *p* < 0.05.

## 3. Results

### 3.1. Characteristics of Study Participants

Table 1 presents an overview of the demographic characteristics of the study participants. The median age of the patients was 51.5 years, with the majority being male (62.5%). The predominant residential setting among the participants was big cities, accounting for 41.1%, while a smaller proportion resided in rural settings (14.1%). More than half of the patients had attained higher education levels, with only a few individuals lacking a high school education. Furthermore, over three-quarters of the participants were gainfully employed. Participants were categorized into four groups based on the time since their initial diagnosis of urological cancer. The first three groups represented consecutive three-year intervals from the time of diagnosis, while the last group encompassed those who had been diagnosed over three years ago. The first three groups each comprised approximately 30% of the patients, whereas the final group was the smallest, consisting of 11.7% of the total participants.

### 3.2. Physical Activity

Table 2 illustrates the stratification of PA levels in relation to the time since diagnosis. Notably, walking was the predominant form of PA among the participants, with no significant differences observed across the groups. However, significant differences were observed in Moderate PA (*p* = 0.01, η^2^ = 0.06) and Vigorous PA (*p* < 0.0001, η^2^ = 0.16), with patients diagnosed within the first year and those diagnosed more than three years ago exhibiting higher levels of PA in both cases. Collectively, those diagnosed within the first year demonstrated the highest overall PA, statistically significant with a moderate effect size (*p* = 0.01, η^2^ = 0.07). This group also had the lowest percentage of inactive individuals (31.4%) and the highest percentage meeting HEPA recommendations (48.6%). In contrast, the group diagnosed between 1 and 2 years ago had the highest percentage of inactive individuals (55.0%) and the lowest HEPA compliance (2.5%). These findings were statistically significant but with a small effect size (*p* < 0.0001, V = 0.19).

Table 3 presents the disparities in PA based on gender and the time since diagnosis. It is evident that gender differences were significant only within the first year following diagnosis. The impact of gender on these differences was moderate for Walking PA (r = 0.36) and Total PA (r = 0.38) but small for Moderate PA (r = 0.19) and Vigorous PA (r = 0.03). Within-gender variations for diagnostic intervals were observed in both men and women for Walking PA, while for the remaining activities, differences existed only among men. Notably, men exhibited a declining trend in PA as the time from diagnosis increased, with a large effect size for all of them. Conversely, women demonstrated an opposite trend, engaging in more Walking PA as the time since diagnosis extended, with a moderate effect size.

Table 4 displays the levels of PA categorized by the time since diagnosis and gender. It is important to highlight that only the men met the criteria for HEPA, while none of the women in any of the periods reached this level of PA. Significant differences emerged only among men (*p* < 0.0001), with a noticeable decline in physically active individuals after the first year following diagnosis and a return to higher activity levels after the third year post diagnosis. Among women, the majority, over 80%, were inactive in the first year following diagnosis, and then the proportions fluctuated from approximately ½ to 2/3; however, these variations did not achieve statistical significance (*p* = 0.30).

### 3.3. Alcohol

Table 5 presents data regarding alcohol consumption. Noteworthy is the relatively high proportion of abstainers, hovering around 50%, in the early periods, which decreases to 20% among those diagnosed with cancer over three years ago. Although, these differences are not statistically significant and exhibit a low effect size. Analysis of the AUDIT questionnaire was conducted solely among individuals reporting alcohol consumption. In this regard, a significant difference in AUDIT scores between diagnostic intervals was observed, with a moderate effect size (*p* = 0.01, η^2^ = 0.12). The highest score was recorded in the initial period, at 4.20 points, still indicative of low-risk drinking. Gender-stratified analysis reveals that in the first two periods, men consumed significantly more alcohol than women. However, analysis for the two latter periods is not feasible due to only one woman reporting alcohol consumption in both. Gender-based differences over time in both cases did not reach statistical significance. It is noteworthy that only two individuals in the entire study, both in the initial period, fell within the category of “Medium risk drinking”, with none classified as “High risk” or higher.

### 3.4. Cigarettes Smoking

Table 6 provides information regarding cigarette smoking among the participants. In each of the time periods, no more than one-third of the subjects reported smoking, and any potential differences were statistically insignificant (*p* = 0.51). Analyses involving the Fagerström questionnaire were conducted solely among individuals admitting to cigarette smoking. Variations in the total points across the diagnostic intervals were on the cusp of statistical significance, with a moderate effect size (*p* = 0.08, η^2^ = 0.12). Gender-based differences were either statistically insignificant or indeterminable due to small sample sizes. Notably, statistically significant within-gender differences, characterized by a large effect size, were observed, with men in the initial period accumulating the highest scores. It is worth noting that only three individuals in the study were classified into the highest category of cigarette dependence.

### 3.5. Multiple Logistic Regression Model

Table 7 presents the results of logistic regression with being inactive according to IPAQ as a dependent variable. The likelihood of being classified as inactive was significantly lower for males, while it increased with age. All other dependencies were statistically insignificant.

## 4. Discussion

### 4.1. Physical Activity

The pivotal role of PA in the promotion of health and well-being among cancer patients is a subject of paramount importance. The treatment regimens for urological cancers typically extend over several months to a year, encompassing modalities such as surgical interventions, chemotherapy, and radiotherapy [26,27]. Throughout this protracted treatment period, patients often confront an array of distressing symptoms, the most frequent one being profound fatigue, with 50–96% of cancer patients encountering it both during and after their cancer treatment [28]. Other side effects may include muscle weakness, episodes of dizziness, bone pain, nausea [29,30], and a range of debilitating psychological issues, such as anxiety (up to 63%), depression (95%), and a pervasive loss of motivation [31].

Studies show that PA may help mitigate both the adversities brought about by cancer treatments and the symptoms associated with the disease itself. Some of the significant benefits include the amelioration of fatigue and muscle weakness, an enhanced capacity to cope with dizziness, a reduction in the experience of bone pain [32,33,34], and an overall improvement in mental health, with substantial alleviation of anxiety and depression and restoration of motivational vigor [35,36,37]. Therefore, it is imperative to educate cancer patients about the advantages that can be derived from engaging in PA throughout and following their treatment.

In the course of our investigation, we analyzed the PA patterns of urological cancer patients, dividing them into four distinct groups based on the duration of time that had transpired since their initial diagnosis. Walking emerged as the predominant form of PA within all the groups, which proves to be consistent with existing research [38,39]. Additionally, walking regimens have exhibited efficacy in the management of treatment-related side effects and the enhancement of physical capabilities within the population of cancer patients [40,41]. Walking’s appeal may lie in its safety, adaptability, and accessibility for people of all backgrounds, without the need for specialized skills or costly equipment [42,43].

Instances of Vigorous MET-m/w were not recorded in our study, which aligns with the existing body of research, where the preference for high-intensity PA among cancer patients typically falls within the range of less than 4% to 6% [44,45]. However, research suggests that intensive exercise interventions can lead to improvements in the physical and psychological well-being of cancer survivors, encompassing areas such as cardiovascular fitness and strength, and the incidence of adverse events associated with high-intensity exercise remains minimal [46]. This should be taken into consideration by medical providers as a foundation for enhancing their educational efforts aimed at enlightening cancer patients about diverse approaches to their PA.

Our study revealed noteworthy disparities in the Total MET-m/w values among research groups, accompanied by a discernible trend: patients exhibited heightened PA levels within the first year following diagnosis (Total MET-m/w = 2826.0) and after a period exceeding three years from diagnosis (693.0), while the intervening years manifested reduced PA levels (462.0 and 629.0). This pattern is similarly evident in the HEPA values, with the most favorable scores observed in groups with patients both during the initial year post diagnosis (48.6%) and beyond three years after diagnosis (33.3%). A worrying observation worth highlighting is the absence of any women in our study who achieved HEPA status. Furthermore, patients characterized as inactive or minimally active displayed the highest scores during the intervening years (42.5–55.0%). 

Although PA and exercise offer notable advantages, these results are in line with other studies showing that a substantial proportion of cancer patients, ranging from 25% to 84%, do not engage in sufficient levels of PA [47,48,49]. Moreover, Fassier et al. demonstrated a comparable trend in their findings: the Total MET-m/w levels were at their lowest after the first year following the diagnosis and subsequently exhibited a steady increase, peaking at the 3-year mark post diagnosis [50]. Numerous studies have consistently noted that the reduction in PA subsequent to a cancer diagnosis is primarily associated with a decrease in vigorous activities [51,52,53]. These findings align with the notion that cancer survivors may encounter greater challenges in meeting recommended levels of PA, a difficulty arising from both the presence of cancer itself and the enduring effects of cancer-related treatments. The upturn in PA levels beyond the three-year mark post diagnosis could be attributed to the amelioration of cancer-related symptoms and the mitigation of treatment-induced side effects. Additionally, it may reflect a desire to resume a regular and healthy lifestyle.

In our research, we observed statistically significant disparities between male and female participants, with males demonstrating notably higher levels of PA, particularly in the initial year following the diagnosis. This concurs with a comprehensive study encompassing a substantial population of 471,760 participants [54], which revealed that male bladder cancer patients exhibited significantly higher levels of PA than female ones. This trend is consistent not only within the context of cancer but also for the general population [55].

### 4.2. Alcohol Use and Abuse

Insufficient research has been conducted to comprehensively analyze alterations in alcohol consumption patterns among individuals subsequent to receiving a cancer diagnosis. Current investigations, while limited in scope, tentatively suggest that approximately 34% to 57% of patients persist in their alcohol consumption habits after the onset of cancer [56,57]. In the context of our study, we observed a notable prevalence of abstinence in the immediate aftermath of diagnosis, with a consistent proportion of approximately 50% refraining from alcohol use during the initial stages. However, this abstinent trend displayed a gradual decline over time, culminating in a proportion of approximately 20% among individuals who had been diagnosed with cancer for a duration exceeding three years.

Allison’s study of cancer patients, with a median post-treatment period of 14 months, revealed a noteworthy increase in the frequency of alcohol consumption as the time elapsed since surgery [58]. These findings are in line with previous research, which suggests an initial decrease in alcohol use arising from the diagnosis, followed by a subsequent and significant upsurge [59]. This initial reduction in alcohol consumption post diagnosis may be associated with the patient’s heightened perception of health risks, although this perception tends to diminish gradually over time [60]. Notably, in a separate study, the percentage of participants who self-reported alcohol consumption appeared to remain consistent regardless of the lengthening interval since their cancer diagnosis [61]. Conversely, in our investigation, we observed that the highest AUDIT scores occurred within the first year after the cancer diagnosis. Subsequently, there was a marked reduction in the AUDIT scores during the 1–3 years post-diagnosis period, followed by a subsequent increase in the AUDIT scores three or more years after the initial diagnosis. The initial decline in alcohol consumption following the cancer diagnosis can also be attributed to the adverse effects of cancer treatments, which may induce symptoms such as nausea and vomiting, thereby reducing the inclination to consume alcoholic beverages.

In our investigation, we observed that male participants consistently achieved significantly higher scores on the AUDIT, particularly in the initial stages following their cancer diagnosis, in comparison to their female counterparts. This finding is in concurrence with established research in the field of oncology, which indicates that male cancer patients tend to exhibit a higher propensity for alcohol consumption and engage in riskier drinking behaviors when compared to women [62,63]. Furthermore, this observation aligns with broader population-based studies that also identify these gender-related disparities in alcohol consumption patterns [64]. Research indicates that the reasoning behind this phenomenon may be due to the fact that individuals identified as adhering to traditional masculine norms, characterized by a propensity for risk-taking and engaging in promiscuous behavior, are more likely to exhibit patterns of heavy drinking. Consequently, the perception of heavy drinking as a stereotypically “masculine” behavior suggests that men conforming to such norms are predisposed to consuming alcohol to intoxication and encountering issues related to alcohol consumption [65].

A wealth of empirical studies consistently underscores the role of alcohol as a risk factor for a multitude of complications. Conspicuously, persistent heavy alcohol consumption during surgery and/or radiation therapy has been empirically linked to heightened susceptibility to treatment-related complications, increased proclivity for cancer recurrence, and a notably diminished rate of survival [66,67]. Individuals classified as alcohol dependent face a significantly heightened risk, ranging from two- to fourfold, of encountering complications during and after cancer surgery [68]. This risk is further exemplified by another study’s findings, which showed that after oropharyngeal tumor resection, intensive care stays were significantly prolonged in patients abusing alcohol [69]. Additionally, an aforementioned study demonstrated that individuals who continued to consume more than 14 alcoholic drinks per week after cancer treatment faced a 50% higher risk of developing second primary tumors (SPTs) [56]. Furthermore, the persistence of alcohol consumption was associated with a more than threefold increase in the risk of developing SPTs, underscoring the substantial consequences of continued alcohol use in this patient group [70]. Moreover, accumulating evidence indicates a potential causative role of alcohol consumption in the development of renal cell carcinoma [71]. 

Notably, our research has unveiled a rather encouraging outcome, with the substantial majority of alcohol consumers demonstrating low-risk drinking patterns. In this context, merely two patients exhibited medium-risk drinking behavior. This observation stands in contrast to the findings of a comprehensive population-based study that investigated alcohol consumption among adult cancer survivors. In that extensive study, a notable proportion, specifically 34.9% of drinking cancer survivors, exceeded the defined limits for moderate drinking, and an additional 21.0% engaged in the practice of binge drinking [61]. It is imperative to underscore the significance of providing patients with comprehensive education concerning the detrimental ramifications of alcohol consumption, particularly within the context of their cancer treatment and recovery.

### 4.3. Cigarette Smoking

A significant proportion of our study participants were non-smokers, and among those who did report smoking, the vast majority were male. As a result, our findings regarding nicotine consumption patterns may not be broadly representative of the larger population of cancer patients. The data obtained in our study do not indicate a clear and statistically significant change in smoking trends over time. However, a similar pattern emerges, akin to the other variables investigated—an initial decline in cigarette smoking between 1 and 2 years post diagnosis followed by a return to previous habits over time. It is also noteworthy that within our study, only three patients exhibited a high level of nicotine dependence. Research shows that the self-reported prevalence of smoking among individuals who have survived cancer varies within the range of 11% to 33% in specific research studies [72,73]. It is consistent with our findings, where 17.1–31.6% of the participants were smokers. 

Research in the general population reveals a noteworthy shift, where the prevalence of smoking among females has exceeded that of males [74]. This shift is supported by compelling evidence indicating that females encounter greater challenges when attempting to cease smoking, attributable to a combination of biological and psychological factors that imply a potentially heightened degree of nicotine addiction among women [75,76]. It has been found that females exhibit an increased probability of familial and relational associations with smoking, encompassing parents, siblings, and romantic partners. They are more inclined to perceive supportive environments for cessation efforts from their social circles. Additionally, if females have friends who smoke, they tend to engage in greater smoking prior to intervention, and they demonstrate diminished motivation and confidence for cessation if a parent smokes. Conversely, males demonstrate an elevated likelihood of diminished cessation motivation and confidence, coupled with a reduced propensity to quit, particularly when they have friends with smoking habits [77]. However, it is noteworthy that existing research concerning specifically cancer patients mirrors the trends observed in our study, wherein males continue to predominate as smokers [78,79]. Research findings indicate that the activation of reward pathways in men appears to be more pronounced in association with smoking when compared to women. This implies that men may engage in smoking primarily for its rewarding effects [80].

The extant body of research pertaining to nicotine dependency levels among cancer patients, apart from those with lung cancer, is notably constrained. Nevertheless, the available research focused on head and neck cancer patients indicates that a substantial proportion, specifically 66.2% of such patients, exhibited high or very high levels of nicotine dependence [81]. Conversely, within our study sample encompassing patients with urological cancer, it was observed that the majority of smokers demonstrated low or moderate nicotine dependency.

The fluctuation in smoking rates among cancer patients, with initial cessation followed by relapse, can be attributed to various factors. Initially, the motivation and interest in smoking cessation expressed by patients with cancer led to lower relapse rates compared to the general population [82]. However, the observed relapse rates among cancer patients can be as high as 60% [83], indicating the complexity of maintaining smoking cessation in this population. Studies have shown that less than half of smoking cancer patients quit smoking after their diagnosis, and only a portion of them receive smoking cessation counseling from their physicians [84]. This highlights the need for more comprehensive and effective support for smoking cessation in cancer care settings.

### 4.4. Future Perspectives

Many PA intervention programs among cancer patients have not fulfilled their motivational function, primarily due to general health disorders, time constraints, and a lack of belief in the significance of PA for health improvement [85]. Encouraging patients to participate in such programs can pose a significant challenge for healthcare professionals [85]. Therefore, it is advisable to adapt these programs according to the individual capacities and reactions of the patients [86]. While a small fraction of intervention programs proved effective, the majority did not yield sustained results in the long term [87]. Key factors for program effectiveness include social support, patient motivation, and meticulous planning [87]. The utilization of the DESIGN procedure has shown efficacy in constructing similar programs [88]. However, evidence for the long-term maintenance of physical activity post-exercise intervention for individuals living with or beyond cancer is limited and inconclusive, emphasizing the need for follow-ups to assess intervention efficacy [87].

Similarly, interventions aimed at reducing alcohol consumption exhibit a comparable ambiguity regarding their long-term effectiveness [89]. Brief alcohol interventions, however, have demonstrated effectiveness [89,90]. A randomized controlled trial by Barticevic et al. indicated that the efficacy of an informational leaflet is similar to a brief intervention conducted by a healthcare professional [91]. This suggests that even the introduction of cost-effective programs, such as well-validated informational leaflets, could significantly impact alcohol reduction, especially if designed for use across various linguistic contexts.

In the context of smoking cessation, behavioral support has proven effective, though various forms of counseling have shown some degree of effectiveness [92]. We particularly highlight the potential of virtual support forms, such as applications [93] or periodic information delivery to patients [94], as these have proven effective without imposing a substantial financial burden on the healthcare system. Therefore, we advocate for interventions distributed over a specific period, during which patients are reminded of the need for lifestyle changes. This could involve the use of pamphlets and information conveyed through communication channels. The establishment of support groups could also prove beneficial.

In summary, adopting a comprehensive, gender-sensitive approach and allocating resources for physical activity, alcohol management, and smoking cessation initiatives could enhance the holistic care of urological cancer patients, especially during critical post-diagnosis phases. 

The initiation of a prospective longitudinal study is crucial for elucidating dynamic changes in health behaviors among cancer patients over time. This investigation is essential for understanding the multifaceted interactions between these behaviors and facilitating the development of targeted interventions and strategies to optimize the well-being and outcomes of individuals undergoing cancer treatment.

The implementation of a longitudinal study is imperative for several key reasons. Firstly, the ability to observe specific patients would eliminate potential variations arising from the individual characteristics of the participants, the specific timing of data collection, and the severity of the disease affecting treatment duration. This approach would provide valuable insights into how individual tendencies evolve, allowing for a more nuanced understanding of the dynamic nature of health behaviors in the context of cancer treatment. By addressing these factors, the longitudinal study design enhances the reliability and validity of the findings, thereby strengthening the foundation for evidence-based interventions and strategies customized to meet the specific needs of cancer patients.

### 4.5. Limitations

This study, while contributing valuable insights, is not without its limitations. The primary limitation pertains to the relatively small sample size, which encompassed 128 urological cancer patients. The makeup of our study cohort primarily consists of individuals residing in urban areas, to some extent mirroring the geographic characteristics of the region; however, there remains an underrepresentation of individuals living in rural areas [95]. It is essential, nonetheless, to contemplate whether this disparity may be attributed to a diminished detectability of urological malignancies among rural residents, stemming from inferior access to diagnostic facilities [96]. Additionally, the gender distribution within our study was skewed, with a majority being male participants, potentially influencing the gender-specific aspects of our results. However, this observation aligns with the demographic profile of urological cancer patients in Poland, where approximately two-thirds are males [1]. Additionally, the predominant representation of participants from big cities raises concerns about the broader applicability of our findings. To address this, future research should aim for a more balanced gender representation and diverse geographical inclusion to enhance the external validity of the results. 

The limited number of smokers in our sample further restricts our ability to draw comprehensive conclusions regarding smoking behaviors among urological cancer patients. 

Another limitation is the focus on a constrained set of lifestyle factors. Incorporating other aspects such as diet, stress levels, and sleep patterns could offer a more comprehensive understanding of post-cancer diagnosis lifestyle changes. It is important to acknowledge that our study relied on self-reported survey data, which may introduce response bias or recall inaccuracies. 

Moreover, our study’s cross-sectional design categorizes patients based on time since diagnosis but does not track individual changes over time. A longitudinal study design would provide more insightful data on lifestyle changes and their impacts, enhancing the robustness and depth of our findings. Finally, the scarcity of existing research specific to urological cancer patients limits our capacity to make direct comparisons with prior studies in this particular context. These limitations should be taken into consideration when interpreting and applying the findings of this research.

## 5. Conclusions

PA plays a crucial role in improving the well-being of urological cancer patients and alleviating symptoms like fatigue, muscle weakness, and psychological distress. Walking was the most prevalent form, consistent with research, while vigorous MET was not observed. Despite the low preference for high-intensity activity, intensive exercise can improve physical well-being. PA levels were highest in the first year and beyond three years post diagnosis, aligning with challenges in maintaining activity after one year.

Limited research examines post-cancer diagnosis alcohol consumption. Existing studies suggest that patients continue to drink, with our study showing a decline in abstinence from diagnosis to three years post diagnosis. The highest AUDIT scores were within the first year, followed by a decrease during the 1–3-year period and an increase after three years. The initial drop in alcohol consumption post diagnosis can be associated with adverse effects of cancer treatments. Male cancer patients, especially in the initial stages following diagnosis, consistently scored higher on the AUDIT.

Our study, with a significant proportion of non-smokers, may not fully represent cancer patients’ nicotine consumption patterns. Existing research shows variable smoking prevalence among cancer survivors (9% to 33%). While there is a notable shift in the general population, with more females smoking, cancer patient research aligns with our study, with males predominating as smokers. Research on nicotine dependency in non-lung cancer patients is limited.

## Figures and Tables

**Table 1:** Characteristics of study participants.

Variables	Total, *N* = 128 [IQR], (%)
**Age**	51.5 [43.5–60.5]
**Gender**	
Male	80 (62.5)
Female	48 (37.5)
**Place of residence**	
Rural area	18 (14.1)
City <50,000 *	23 (18.0)
City 50,000–100,000 *	34 (26.6)
City 100,000+ *	53 (41.1)
**Education**	
Primary	2 (1.6)
Vocational	11 (8.6)
Secondary	50 (39.1)
Higher	65 (50.8)
**Employment situation**	
Employed	99 (77.3)
Unemployed	21 (16.4)
Retired	4 (3.1)
Studying	4 (3.1)
**Time since diagnosis**	
<1 year	35 (27.3)
1–2 years	40 (31.3)
2–3 years	38 (29.7)
>3 years	15 (11.7)

Note: *N* is the number of observations; * Number of inhabitants.

**Table 2:** Comparison of physical activity MET and levels by time since the diagnosis.

	<1 Year, *N* = 35	1–2 Years, *N* = 40	2–3 Years, *N* = 38	>3 Years, *N* = 15	*p*-Value	Effect Size
**Physical activity MET-m/w**						
Walking MET-m/w	693.0 ^1^ [346.5–1386.0 ^2^]	429.0 [346.5–693.0]	462.0 [297.0–1039.0]	462.0 [346.5–924.9]	*p* = 0.32	η^2^ = 0.00
Moderate MET-m/w	180.0 [0.0–720.0]	0 [0.0–210.0]	0 [0.0–240.0]	240.0 [0.0–560.0]	*p* = 0.01	η^2^ = 0.06
Vigorous MET-m/w	0 [0.0–1440.0]	0 [0.0–0.0]	0 [0.0–0.0]	0 [0.0–2000.0]	*p* < 0.0001	η^2^ = 0.16
Total MET-m/w	2826.0 [471.0–3672.0]	462.0 [346.5–993.0]	629.0 [438.0–1386.0]	693.0 [346.5–4266.0]	*p* = 0.01	η^2^ = 0.07
**Physical activity level**						
Inactive	11 (31.4 ^3^)	22 (55.0)	17 (44.7)	6 (40.0)	*p* < 0.0001	
Minimally active	7 (20.0)	17 (42.5)	17 (44.7)	4 (26.7)		V = 0.19
HEPA	17 (48.6)	1 (2.5)	4 (10.5)	5 (33.3)		

Note: ^1^—median; ^2^—interquartile range; ^3^—column percentage.

**Table 3:** Comparison of physical activity MET by time since diagnosis and gender.

	<1 Year, *N* = 35	1–2 Years, *N* = 40	2–3 Years, *N* = 38	>3 Years, *N* = 15	*p*-Value ^1^	Effect Size
**Walking MET-m/w**						
Male	1180.0 ^3^ [693.0–2772.0 ^4^]	462.0 [346.5–693.0]	462.0 [297.0–1039.0]	462.0 [346.5–924.0]	*p* = 0.002	η^2^ = 0.16
Female	330.0 [0.0–346.5]	396.0 [346.5–693.0]	346.5 [297.0–577.5]	346.5 [231.0–693.0]	*p* = 0.03	η^2^ = 0.13
*p*-Value ^2^	*p* < 0.0001	*p* = 0.64	*p* = 0.20	*p* = 0.31		
Effect size	r = 0.36	r = 0.04	r = 0.11	r = 0.09		
**Moderate MET-m/w**						
Male	300.0 [50.0–1320.0]	0.0 [0.0–0.0]	120.0 [0.0–180.0]	420.0 [0.0–1000.0]	*p* = 0.004	η^2^ = 0.14
Female	80.0 [0.0–240.0]	0.0 [0.0–240.0]	0.0 [0.0–240.0]	0.0 [0.0–0.0]	*p* = 0.47	η^2^ = 0.00
*p*-Value	*p* = 0.04	*p* = 0.27	*p* = 0.36	*p* = 0.08		
Effect size	r = 0.19	r = 0.08	r = 0.08	r = 0.15		
**Vigorous MET-m/w**						
Male	800.0 [0.0–2040.0]	0.0 [0.0–0.0]	0.0 [0.0–0.0]	0.0 [0.0–2200.0]	*p* < 0.0001	η^2^ = 0.33
Female	0.0 [0.0–0.0]	0.0 [0.0–0.0]	0.0 [0.0–0.0]	0.0 [0.0–0.0]	*p* = 0.52	η^2^ = 0.00
*p*-Value	*p* = 0.0006	*p* = 0.68	*p* = 0.21	*p* = 0.23		
Effect size	r = 0.03	r = 0.02	r = 0.11	r = 0.11		
**Total MET-m/w**						
Male	3273.0 [2599.5–6453.0]	462.0 [346.5–693.0]	636.0 [438.0–1546.5]	882.0 [404.25–4799.0]	*p* < 0.0001	η^2^ = 0.28
Female	330.0 [0.0–586.5]	636.0 [346.5–1117.5]	586.5 [577.5–857.5]	346.5 [231.0–693.0	*p* = 0.08	η^2^ = 0.09
*p*-Value	*p* < 0.0001	*p* = 0.41	*p* = 0.55	*p* = 0.11		
Effect size	r = 0.38	r = 0.07	r = 0.05	r = 0.14		

Note: ^1^—comparison between durations intervals; ^2^—comparison between genders; ^3^—median; ^4^—interquartile range.

**Table 4:** Comparison of physical activity levels by time since diagnosis and gender.

	<1 Year, *N* = 35	1–2 Years, *N* = 40	2–3 Years, *N* = 38	>3 Years, *N* = 15	*p*-Value	Effect Size
**Physical activity level—male**						
Inactive	2 (8.3 ^1^)	12 (63.2)	10 (40.0)	4 (33.3)	*p* < 0.0001	
Minimally active	5 (20.8)	6 (31.6)	11 (44.0)	3 (25.0)		V = 0.24
HEPA	17 (70.8)	1 (5.3)	4 (16.0)	5 (41.7)		
**Physical activity level—female**						
Inactive	9 (81.8)	10 (47.6)	7 (53.9)	2 (66.7)	*p =* 0.30	
Minimally active	2 (18.2)	11 (52.4)	6 (46.2)	1 (33.3)		V = 0.16
HEPA	0 (0.0)	0 (0.0)	0 (0.0)	0 (0.0)		

Note: ^1^—column percentage.

**Table 5:** Comparison of alcohol use and abuse by time since diagnosis and gender.

	<1 Year, *N* = 35	1–2 Years, *N* = 40	2–3 Years, *N* = 38	>3 Years, *N* = 15	*p*-Value ^1^	Effect Size
**Do you ever drink alcohol?**						
Yes	15 (42.9 ^5^)	22 (55.0)	21 (55.3)	12 (80.0)	*p* = 0.12	V = 0.12
No	20 (57.1)	18 (45.0)	17 (44.7)	3 (20.0)		
**AUDIT points**	4.20 ^3^ (2.02–6.38 ^4^)	2.00 (1.57–2.43)	3.33 (2.42–4.25)	3.67 (2.84–4.49)	*p* = 0.01	η^2^ = 0.12
**AUDIT points**						
Male	4.92 ^3^ (2.31–7.52 ^4^)	2.50 (1.86–3.14)	3.35 (2.39–4.31)	3.91 (3.21–4.61)	*p* = 0.12	η^2^ = 0.05
Female	1.33 (0.0–2.78)	1.40 (1.03–1.77)	3.0 (3.0–3.0)	1.0 (1.0–1.0)	*p* = 0.26	η^2^ = 0.09
*p*-Value ^2^	*p* = 0.045	*p* = 0.01	*p* = 1.00	*p* = 1.00		
Effect size	r = 0.52	r = 0.54	r = 0.00	r = 0.00		
**AUDIT level**						
Low risk drinking	13 (86.7 ^5^)	12 (100.0)	21 (100.0)	12 (100.0)	*p* = 0.06	V = 0.19
Medium risk drinking	2 (13.3)	0 (0.0)	0 (0.0)	0 (0.0)		

Note: Abstainers were excluded from the analysis; ^1^—comparison between durations intervals; ^2^—comparison between genders; ^3^—mean; ^4^—95% CI; ^5^—column percentage.

**Table 6:** Comparison cigarette smoking by time since diagnosis and gender.

	<1 Year, *N* = 35	1–2 Years, *N* = 40	2–3 Years, *N* = 38	>3 Years, *N* = 15	*p*-Value ^1^	Effect Size
**Do you smoke cigarettes?**						
Yes	6 (17.1 ^5^)	12 (30.0)	12 (31.6)	4 (26.7)	*p* = 0.51	V = 0.08
No	29 (82.9)	28 (70.0)	26 (68.4)	11 (73.3)		
**Fagerström points**	4.83 ^3^ (2.80–6.87 ^4^)	2.42 (1.58–3.25)	4.17 (2.54–5.79)	3.75 (0.47–7.03)	*p* = 0.08	η^2^ = 0.12
**Fagerström points**						
Male	5.40 ^3^ (3.52–7.28 ^4^)	2.29 (1.41.–3.17)	4.80 (3.16–6.45)	3.75 (0.47–7.03)	*p* = 0.02	η^2^ = 0.30
Female	2.00 (2.00–2.00)	2.60 (0.34–4.86)	1.00 (1.00–1.00)	0.0 (0.0–0.0)	*p* = 0.36	η^2^ = 0.00
*p*-Value ^2^	*p* = 1.00	*p* = 0.93	*p* = 0.10	*p* = 1.00		
Effect size	r = 0.00	r = 0.02	r = 0.48	r = 0.00		
**Fagerström level**						
Low dependence (0–3 points)	1 (16.7 ^5^)	10 (83.3)	4 (33.3)	1 (25.0)		
Moderate dependence (4–6 points)	3 (50.0)	2 (16.7)	7 (58.3)	3 (75.0)	*p* = 0.03	V = 0.19
High dependance (7–10 points)	2 (33.3)	0 (0.0)	1 (8.3)	0 (0.0)		

Note: Non-smokers were excluded from the analysis; ^1^—comparison between durations intervals; ^2^—comparison between genders; ^3^—mean; ^4^—95% CI; ^5^—column percentage.

**Table 7:** Multiple logistic regression model with being inactive according to IPAQ as a dependent variable.

Variables	Estimate	Standard Error	aOR	95% CI for aOR	*p*-Value
**Intercept**	−2.780	1.025	0.062	0.008–0.463	0.007
**Age**	0.039	0.015	1.040	1.009–1.072	0.0120
**Gender**					
Female	Reference	Reference	Reference	Reference	Reference
Male	−0.956	0.375	0.385	0.184–0.802	0.0108
**Place of residence**					
City 100,000+ *	Reference	Reference	Reference	Reference	Reference
City 50,000–100,000 *	0.303	0.444	1.354	0.568–3.232	0.4941
City <50,000 *	−0.405	0.533	0.667	0.234–1.895	0.4469
Rural area	1.114	0.573	3.048	0.990–9.378	0.0520
**Time since diagnosis**					
<1 year	Reference	Reference	Reference	Reference	Reference
1–2 years	0.981	0.483	2.667	1.034–6.877	0.0424
2–3 years	0.569	0.489	1.766	0.677–4.605	0.2446
>3 years	0.375	0.641	1.455	0.414–5.105	0.5586
**Do you drink alcohol?**					
No	Reference	Reference	Reference	Reference	Reference
Yes	−0.336	0.359	0.714	0.354–1.442	0.3481
**Do you smoke cigarettes?**					
No	Reference	Reference	Reference	Reference	Reference
Yes	0.344	0.402	1.410	0.642–3.100	0.3923

Note: aOR—adjusted odds ratio. * Number of inhabitants.

## Data Availability

The datasets used and analyzed during the current study are available from the corresponding author upon reasonable request.
